# Collaborative Governance for Integrated Care: Insights from a Policy Stakeholder Dialogue

**DOI:** 10.5334/ijic.4684

**Published:** 2020-02-11

**Authors:** Dara Gordon, Sandra McKay, Gregory Marchildon, R. Sacha Bhatia, James Shaw

**Affiliations:** 1 Institute for Health System Solutions and Virtual Care, Women’s College Hospital, Toronto, Ontario, CA; 2 Research & Education Department, VHA Home HealthCare, CA; 3 Department of Physical Therapy, Faculty of Medicine, University of Toronto, Toronto, Ontario, CA; 4 North American Observatory on Health Systems and Policies, CA; 5 Institute of Health Policy, Management and Evaluation, University of Toronto, Toronto, Ontario, CA; 6 Institute for Clinical Evaluative Sciences, CA; 7 Faculty of Medicine, University of Toronto, Toronto, Ontario, CA

**Keywords:** integrated care, implementation science, health care management, collaborative governance

## Abstract

**Introduction:**

Integrated care is a goal of many health care systems. However, operationalizing and implementing integrated care remains challenging especially in continuously evolving policy environments. We report on a policy symposium held in 2017 focused on operationalizing a particular integrated care policy in the context of policy evolution in Ontario, Canada.

**Methodology:**

Forty-five participants attended the symposium including government employees, health care leaders, researchers, clinicians, and patient representatives. The symposium included presentations from representatives of each group and breakout sessions. Two trained observers recorded observational field notes.

**Results:**

We report four recommendations and fourteen sub-recommendations which arose regarding the implementation of the policy. We highlight four important tensions which characterize challenges regarding its implementation, and discuss the recommendations in the context of Collaborative Governance.

**Discussion:**

We outline how the recommendations could be strengthened by collaborative governance and identify where this framework could support governance and leadership challenges associated with implementing integrated care. We describe the unique challenges posed by working towards these goals in an evolving policy environment.

**Conclusion:**

We draw on collaborative governance to generate insights for leaders implementing integrated care and conclude by addressing the importance of maintaining collaborative governance initiatives under circumstances of unstable policy environments.

The goal of achieving integrated care increasingly characterizes the efforts of health systems around the world to improve effectiveness, efficiency and outcomes, yet the implementation of integrated care remains an extremely complex challenge. Although large multi-national projects have provided early understandings into the optimal ways to implement more integrated models of care [1,2], insights into strategies to navigate the political and managerial demands of implementing change are difficult to generate. In this paper, we report on a policy stakeholder dialogue that took place in 2017 and was focused on operationalizing a particular policy in Ontario, Canada intended to promote better local integration of health services. We report the overall recommendations arising from the dialogue, highlight tensions that arose during the discussion, and describe new policy developments that have occurred in Ontario since the introduction of the policy in question. We draw on the theory of collaborative governance to generate insights for managers and leaders intending to implement more integrated models of care under circumstances of unstable policy environments [3]. We conclude with comments on the importance of establishing a vision for the maintenance of collaborative initiatives even in circumstances where institutional realities might be changing.

## Background

The term “integrated care” has been defined and used in different ways but is often understood as representing the effort to improve the quality of care for individual patients, service users and caregivers by ensuring that services are well coordinated around their needs across different care environments [4]. In his seminal paper defining integrated care, Leutz suggested that integration is required at many levels: “the means of integration include joint planning, training, decision making… information systems, purchasing, screening and referral, care planning, benefit coverage, service delivery, monitoring, and feedback” [5]. A more recent review of 7 integrated care programs in Australia, New Zealand, the Netherlands, the UK, Sweden, USA, and Canada, showed that all involved bottom-up innovation driven by local needs, and highlighted five key components of integrated care. These were: (1) a single point of entry, (2) holistic care assessments, (3) comprehensive care planning, (4) care co-ordination and (5) a well-connected provider network [4]. The basic consensus in the integrated care literature appears to be that without integration at various levels and of various types (e.g., clinical, normative, functional, etc), “all aspects of health care performance [would] suffer. Patients get lost, needed services fail to be delivered, or are delayed, quality and patient satisfaction decline[s], and the potential for cost-effectiveness diminishes” [6,7].

### A Focus on Governance

In our paper we are focused on the governance challenges faced by managers and other health care leaders as they work to foster more integrated health care delivery in the face of changing policy environments. The term governance has been used in a wide variety of ways across disciplines [8]. In discussions of public services, the term has largely been used to represent a shift away from historical, heavily bureaucratic forms of government toward a growing emphasis on decentralized decision-making guided by performance measurement and related sanctions [9,10]. We treat governance as the interface between the system-wide rules of governmental policy and the strategic decisions of organizational leaders, constituting a set of organizational policies and practices that construct the boundaries around acceptable organizational activity. In this way, governance includes the activities associated with ensuring an organization abides by governmental policy (such as achieving performance targets), and also the activities associated with carrying out its independently defined strategy (such as engaging in formal inter-organizational partnerships).

**Figure 1:**
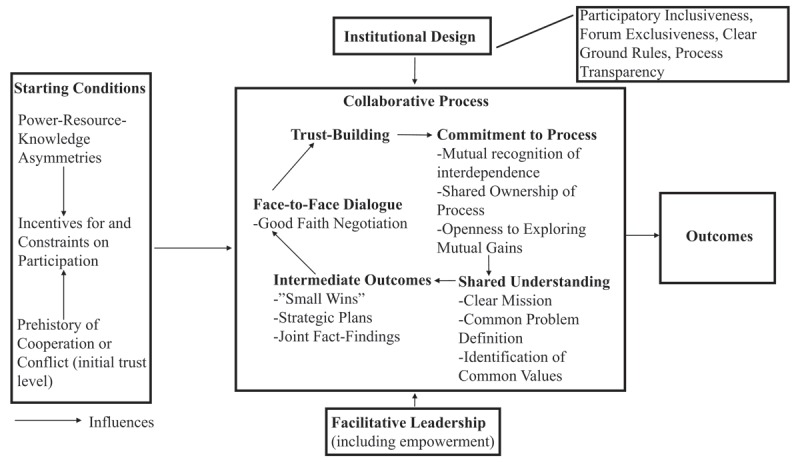
Ansell and Gash (2008) Model of Collaborative Governance (Reproduced with permission).

We frame our analysis of governance issues for integrated care specifically in relation to the framework of Collaborative Governance [3,11]. Ansell and Gash (2008) presented a model of collaborative governance that represents the synthesis of a wide range of existing literature addressing the topic of collaborative governance from various perspectives (see Figure 1, reproduced with permission). The model outlines a robust framework for collaborative governance which specifies the conditions, inputs, and the iterative core process necessary for successful collaboration between multi-sector stakeholders from public and private agencies working to address complex policy reform initiatives [3]. The collaborative governance framework provides an ideal approach to understanding the governance challenges associated with efforts to promote more integrated health and social care delivery.

Although more recent work on collaborative governance has provided an updated model of this concept [11,12], we structured our analysis using Ansell and Gash’s (2008) description. The primary reason for this is that the concept of a collaborative governance “regime” did not appear to resonate with our substantive domain of inquiry. Emerson et al [11] explain that a collaborative governance regime is central to their modified framework, and should be understood as “the particular mode of, or system for, public decision making in which cross-boundary collaboration represents the prevailing pattern of behavior and activity.” (p. 6). We did not begin our analysis assuming that cross-boundary collaboration was a prevailing pattern of behavior, but on the contrary, wanted to use the collaborative governance framework to specify where opportunities would exist to enhance the collaborative decision-making practices of those involved in implementing integrated care policy. As such, we used the more basic descriptive framework of Ansell and Gash [3], which outlines the particular activities and institutional arrangements that could promote more collaborative governance activity at the outset of an implementation initiative.

Four broad concepts are outlined in Ansell and Gash’s paper: starting conditions, institutional design, leadership, and collaborative process [3]. The authors view the collaborative process concepts as the core features of the model and describe the starting conditions, institutional design, and leadership influences as being critical contributions to sustainable collaboration. One further benefit of Ansell and Gash’s collaborative governance model is its relevance to the phase of collaboration *building*, as opposed to the ongoing maintenance of collaborative networks [3]. This point further illustrates its relevance to the organizational challenges of implementing changes in organizational practices related to adopting integrated care. In this section, we provide a brief overview of each element of the collaborative governance model.

### Starting Conditions

In the model, starting conditions “set the basic level of trust, conflict, and social capital that become resources or liabilities during collaboration”. The authors synthesized the starting conditions, which heavily influence the resulting cooperation among stakeholders, into three influences: (a) imbalances between the resources or power of different stakeholders, (b) the incentives that stakeholders have to collaborate, and (c) the past history of conflict or cooperation among stakeholders.

### Institutional Design

Institutional design sets the basic ground rules under which collaboration takes place [3] including the protocols for collaboration, which are critical for the procedural legitimacy of the collaborative process [3]. Collaborative governance is positioned in the model as requiring inclusivity of any stakeholder group that is involved, and transparency about collaboration agreements and processes helps remind stakeholders that the process is fair, equitable and open [3].

### Facilitative Leadership

Facilitative leadership is described as providing essential mediation and facilitation for the collaborative process since it brings stakeholders together and can steer them through challenging components of the collaborative process. This type of leadership also facilitates empowerment of stakeholders and supports weaker stakeholders as it can produce a “balance of power” and can help stakeholders explore possibilities for mutual gain [3].

### The Collaborative Process

The collaborative process itself is iterative and involves stages of communication, trust, commitment, understanding, and outcomes. Ansell and Gash (2008) describe this process as consensus-oriented and allowing for direct dialogue, which is necessary for stakeholders to identify opportunities for mutual gain. A lack of trust among stakeholders is often a common starting point with regards to the collaborative process, especially when there has been a prehistory of antagonism. Ansell and Gash (2008) suggest that good collaborative leaders recognize early on that they must build trust among stakeholders or they will risk efforts of some stakeholders to manipulate the process.

Stakeholders’ level of commitment to collaboration is a key influence in explaining success or failure. Weak commitment from public agencies to collaboration, particularly at the senior level, is problematic. Engaging in collaborative governance shifts the “ownership” of decision-making from the agency to the stakeholders involved. Shared understanding among these stakeholders is related to defining a common mission, purpose, objectives or aims. Its ultimate goal is to come to agreement on a definition of the problem. Ansell and Gash explain that “collaboration is more likely to occur when the possible purposes and advantages of collaboration are relatively concrete” and can result in intermediate outcomes or “small wins” [3]. Tangible outputs are essential for building the momentum of the on-going collaboration.

Collaborative governance is a model that outlines in some detail the many challenges associated with promoting and institutionalizing collaboration between health-related organizations. The historical imbalances in funding and power between physician-focused organizations such as hospitals and nursing/rehabilitation-focused organizations such as home health care is a widely acknowledged tension in many health systems. How this and other tensions can be better understood, agreed upon, and overcome by individuals and organizations intending to work more closely together to provide better care has been an under-represented challenge in the literature on integrated care [13]. In our paper, we focus specifically on identifying potential strategies to enable better inter-organizational collaboration through collaborative governance mechanisms, particularly under circumstances of evolving policy environments.

## The Policy Setting: Ontario, Canada

Ontario is the largest Province in Canada, with a population of over 13 million people, with two urban centres containing over 1 million people, and a number of smaller urban centres dispersed over a very large geographic area. Recent estimates suggest that 59.6% of the population of Ontario lives in urban environments, 9–13% live in small to medium suburban environments, and 18.7% in rural and remote environments [14]. Despite efforts to improve integration, the health care system in the province of Ontario remains fragmented. Ontario, like provinces in the rest of Canada, operates a publicly funded, single-payer health care system for hospital, diagnostic and medical care services in addition to funding long-term care, home care, and limited pharmaceutical coverage. Within this system, most hospitals as well as other health care organizations and physicians are private entities that offer publicly funded services. The health system structure often leads to care being delivered in a highly siloed manner, with little coordination between health care providers, hospitals, home and community care, and other social services.

In the end of 2016, the Ontario government passed *Bill 41: An Act to amend various Acts in the interest of patient-centred care* (referred to simply as Bill 41 or Patients First in this paper) to improve integration of services and service delivery. The Bill outlined several key focus areas, including: (1) effective integration of services and greater equity, (2) timely access to, and better integration of, primary care and (3) more consistent and accessible home and community care. There was widespread recognition following the introduction of this policy that its implementation would pose a number of important challenges. This recognition was the primary motivation for the symposium that is the focus of this paper, described in more detail in the next section.

Bill 41 was a complex piece of legislation amending a variety of existing policies, but two features of the policy are key to its central objective of achieving more integrated, patient-centered care. First, the policy mandated the organizational integration of two previously existing groups of agencies, wherein the 14 Community Care Access Centres (public arms-length agencies responsible for commissioning home care services from a range of private home health care provider agencies) were to be absorbed by the 14 Local Health Integration Networks (LHIN, responsible for commissioning and integrating health care services in local regions). The purpose of this change was to reduce bureaucracy and streamline local health system planning, theoretically enabling more efficient, integrated care in each region.

Second, the policy mandated the establishment of clearly defined sub-regions within each LHIN, such that local level data could be used to plan a complete scope of services required to meet the population health needs of more clearly defined local populations. Sub-regions would contain a median population size of 140,000 [15]. These sub-regions would work with existing integrated care programs and health care organizations to ensure that local communities’ needs are being met through the delivery of integrated health and social care. The sub-regions did not come along with new mechanisms of paying for care. Instead, they were assigned new managerial roles that would sit within the LHIN as regional health system planners, providing leadership for various initiatives related to ensuring more coordinated care for people with the most complex health needs. In this way, the initiative was not about integrating budgets or integrating formal administrative structures at the organizational level. It was about using strategies of local planning to ensure that necessary services were working together in more coordinated ways for the patients most in need.

In June 2018, a Provincial election resulted in a change in governing party, raising questions about the interest of the new majority government in persisting with the recent changes made to health policy by the previous government in 2016. This uncertainty posed important challenges to the effort to implement integrated care, and in February 2019, new legislation was introduced that proposed further substantial changes to the health system. This new legislation, titled *Bill 74: An Act concerning the provision of health care, continuing Ontario Health and making consequential and related amendments and repeals*, explicitly addressed integrated care.

The two primary features of the policy were the establishment of a single agency responsible for several health system planning functions, and a new approach to providing funding for “integrated care delivery systems” that were loosely based on the Accountable Care Organization model arising from the United States of America. These integrated care delivery systems are intended to serve populations of approximately 350,000 people. These integrated care delivery systems are now referred to as “Ontario Health Teams”, and will include a shared funding envelope for a range of services including primary care, mental health, home care, long term care and community services. Groups of organizations will develop applications to receive funds in this new way, and if successful, will then make collaborative decisions about how to spend those funds to better meet the needs of the communities in which they are situated. The demand to establish collaborative groups of organizations in Ontario Health Teams is distinct from the local collaborative arrangements that characterized “sub-regions” arising from Bill 41 as described in the previous section. As a result, previous efforts to better coordinate care around the needs of patients are at risk as organizations develop plans for more grandiose integration ambitions.

This brief description of recent health policy history in Ontario illustrates a number of changes to the structure of the health system specifically in relation to efforts to institutionalize integrated care in the province. The changes to services to be funded in a decentralized manner, size of population to be cared for in an integrated way, specific funding strategies, and forms of support provided to enable integration have changed substantially over a three year period. During this same period of time, organizational leaders and health care providers have been expected to persevere in developing and sustaining the inter-organizational relationships required to enact more integrated approaches to care. This policy context motivates the primary question we pose in this paper: What can organizational leaders learn from collaborative governance to strengthen their approaches to supporting integration initiatives in circumstances of policy evolution?

What are the challenges faced by organizational leaders working to implement integrated care in circumstances of policy evolution, and what collaborative governance strategies might be employed to enable more stable approaches to integrated care in such circumstances?

## Methods

In March of 2017, the Women’s College Hospital Institute for Health System Solutions and Virtual Care (WIHV; an applied health systems research and innovation institute) co-hosted a stakeholder dialogue symposium, along with VHA Home Health Care (a home health care delivery organization) and the North American Observatory on Health Systems and Policies (a comparative health policy research institute) to discuss local-level strategies for implementing integrated care in Ontario, Canada. This symposium was explicitly planned and carried out to address the challenge of implementing the integrated care components of Bill 41.

The symposium engaged a diverse group of key stakeholders including patients and caregiver representatives, clinicians, leaders from health service organizations, leaders from homecare organizations, health system researchers, executives and managers from LHINs and hospitals, as well as municipal and provincial government representatives. Senior officials from both the political decision-making and bureaucratic offices of the provincial government overseeing the operationalization and implementation of this specific Bill were present.

**Figure 2:**
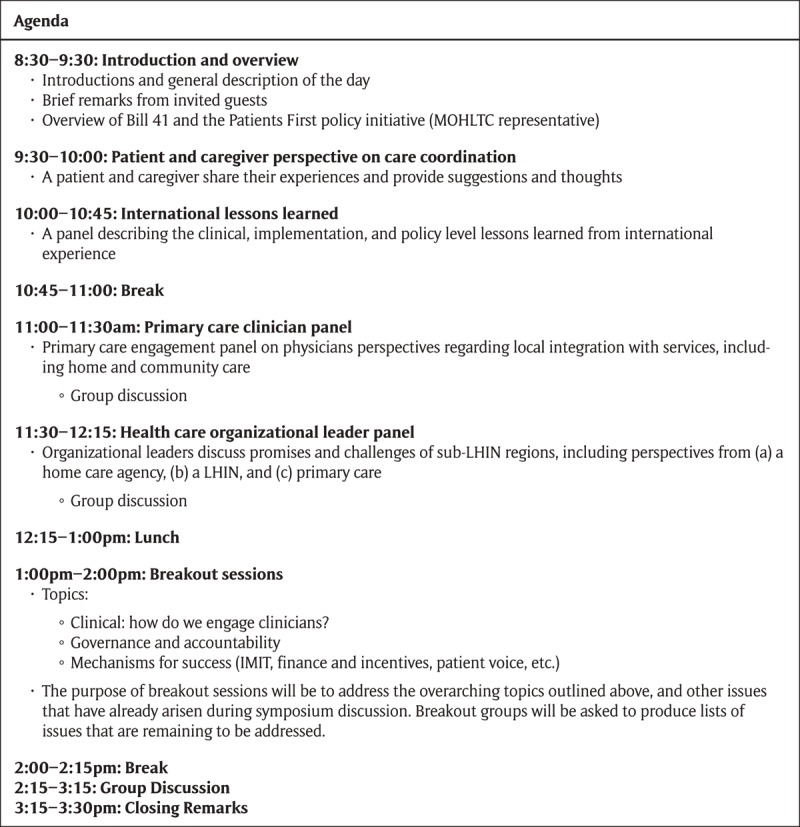
Symposium Agenda.

Forty-five attendees were present at the symposium, including government employees (n = 7), health care organizational leaders (n = 12), researchers (n = 10), clinicians (n = 6), patient and caregiver representatives (n = 3), and other stakeholders (n = 7). The symposium was conducted under Chatham House Rules, meaning that the identities of the symposium participants were not recorded in order to help promote honest, productive dialogue. The symposium included structured presentations from representatives of each group, and breakout sessions focused on brainstorming related to key topics addressed in Bill 41. The discussion at the symposium was encouraged to be honest and forthcoming about the real challenges that would be faced in operationalizing the policy in practice. An agenda can be found in Figure 2. Observational field notes were recorded by two trained observers throughout the day and were synthesized into one final set of notes. Observational notetaking followed the methods outlined by Hammersley and Atkinson (2007), focusing on capturing as much as possible the actual content of dialogue throughout the day [16].

## Analysis

Our analysis proceeded in three phases. First, we sought to identify basic descriptive themes from the symposium discussion that would represent the important points raised by stakeholders participating in the symposium dialogue. These themes are presented as the recommendations arising from the symposium, and are intended to be as closely aligned with the actual discussion held at the symposium as possible. The recommendations came directly from stakeholder participants. In a second phase of analysis, we identified the most important tensions raised by stakeholder participants in the workshop discussion. A number of tensions arose throughout the day, but we selected four to highlight in particular. The four tensions we highlight are those that were most frequently raised and generated the most extensive dialogue about the potential challenges related to implementing integrated care policy. Our effort to identify recommendations and tensions drew specifically on the work of Braun and Clarke (2006) providing guidance for the identification of semantic themes in qualitative data (i.e., those themes that are most practically significant for the research question of focus) [17]. The final phase of our analysis was to relate the recommendations that arose directly to the collaborative governance framework of Ansell and Gash [3]. We did so to identify where the effort to explicitly incorporate a collaborative governance approach could contribute unique insights into the governance and leadership challenges associated with implementing integrated care policy.

## Results

The symposium dialogue was characterized by multiple and sometimes conflicting stakeholder views, as representatives of different sectors brought their different interests and concerns to bear in discussion about the implementation of Bill 41 and integrated care more generally. In this results section we first present the recommendations that arose directly from stakeholder dialogue, and then what we understood to be the most important tensions. We relate the recommendations directly to the collaborative governance framework in the discussion section.

## Stakeholder Dialogue Recommendations

Four recommendations and fourteen related sub-recommendations emerged from our analysis overall (listed in **Box 1**). Recommendation 1 relates to developing strategies for governance and accountability that can help to promote a more integrated health system that focuses on the needs and wishes of patients and families. This theme was informed by the clear recognition among symposium participants that existing accountability structures can discourage stronger collaboration between organizations. A number of suggestions were put forward during the dialogue, focused on thinking more creatively about the links between governance structures and the actual delivery of patient care. Challenges persist in this domain related to bureaucratic red tape that prevents collaboration between healthcare providers and social service agencies such as housing providers from working more closely together to serve the same patients. For this reason, governance and accountability structures which incentivize collaboration between health and social care providers are needed.

**Box 1: Recommendations arising from Thematic Analysis of Policy Symposium**

**Recommendation #1: Enhance approaches to governance and accountability at the LHIN and sub-region levels to promote a more integrated patient experience of the health care system**
1.1 Encourage shared accountability arrangements between health care delivery organizations wherever possible, in order to enable more integrated patient experiences of the health care system.
1.2 Establish clinician-level accountability mechanisms for more integrated care.
1.3 Develop incentives to build collaborative relationships with non-health system stakeholders, in order to connect patients with all the services they need.
**Recommendation #2: Establish metrics and measurement strategies that provide a clear picture of quality across the continuum of care, and reflect the perspective of patients, families and providers**
2.1 Patients and caregivers should be systematically engaged to help co-design priority metrics that can be used to guide the implementation of Patients First.
2.2 Build health care providers perspectives and experiences into the evaluation of Patients First.
2.3 Enable provider and manager access to performance data relevant to their local level of care delivery.
**Recommendation #3: Leverage the sub-regions to enable health care providers to develop, scale and spread innovative strategies of care delivery**
3.1 Identify and share best practices for engaging health care providers in the local development of innovative initiatives, including care coordinators.
3.2 Build clinical leadership at the sub-region level.
3.3 Streamline administrative functions to make innovation easier.
3.4 Build on innovative funding models that promote innovation, and particularly those in the areas of digital and mobile health.
3.5 Develop a provincial communications plan that emphasizes provider opportunities for innovation.
**Recommendation #4: Continue to engage patients and caregivers as central partners in health system planning**
4.1 Continue to enable patient and caregiver engagement at the LHIN and sub-LHIN level.
4.2 Support training and capacity development of patients and caregivers.
4.3 Develop a communications strategy specifically directed to patients and the public that tells the story of how the health care system is changing, why, and what will be different for them as users of the system.

The second recommendation relates to identifying relevant metrics in ways that can leverage the knowledge available within the health system. This recommendation stems from the notion that effective quality improvement and health system change relies on the availability and use of accurate data that is relevant at the sub-region level. At present, collecting this data at local, population-specific levels is ad-hoc and inconsistent. Participants suggested that more careful thought was required when developing these metrics, as they provide a fundamental incentive driving organizational action in health and social care.

The third recommendation was developed based on the recognition that health system innovation is dependent on those working at the local-level, and therefore relates to the strategies needed to leverage the creativity and interest of health care providers and managers to enable innovation. Framed in terms of strategies to promote the scale and spread of innovations, this theme focused on topics such as the sharing of best practices and removing administrative barriers which often prevent clinician-driven adoption of innovations. The development of innovative funding models which incentivize providers to adopt digital health tools in their practices is a prominent example of this endeavor.

The final recommendation is related to a continued commitment to patient and caregiver engagement throughout all of the other processes. Although this is being increasingly recognized in the planning of policy by the Government of Ontario, participants voiced their interest in ensuring that patients and caregivers were the central consideration of policy and planning at the provincial and local levels.

## Tensions in the Implementation of Integrated Care Policy

### Tension 1: Sharing Accountability across Organizations

One of the more controversial suggestions arising from the symposium is presented in Recommendation 1.1. This recommendation encourages shared accountability arrangements between health care delivery organizations wherever possible, in order to enable more integrated patient experiences of the health care system. Many organizations in the current health care landscape in Ontario report to independent boards of directors, based on activities identified as strategically important at the organizational level. However, there was widespread understanding at the symposium that effective integration of care in Ontario requires the coordinated efforts of organizations working toward the same goal. One innovative approach to accomplish such coordinated action is to hold organizations accountable for goals that require collaboration to achieve, such as population-level metrics at the sub-region level (e.g., reducing the frequency of hospitalization caused by exacerbations of certain chronic diseases). Despite an apparent consensus that shared accountability mechanisms would be extremely helpful for efforts to promote more integrated care in local areas, symposium participants expressed skepticism about whether this is a realistic goal for health policy.

### Tension #2: Establishing Standards versus Standardizing Practices

One of the primary purposes of structuring the provincial health system into smaller geographic regions is to enable local leaders to identify important local needs and generate novel approaches to address those needs. This represents the promotion of innovation in integrated care at the local level, based on the effort to implement models of care that make the most sense at the local level. However, at the symposium this viewpoint was countered by those emphasizing the importance of standardized practices across the entire province in order to minimize unexplained variations in care. Like many health systems in high-income countries, the health system in Ontario has substantial unexplained variations in care [18], including in models of integrating home care with primary and hospital care. In order to promote equity, those holding this view suggested that more effort should be expended to spread and scale models of integrated care that are shown to work. Differing beliefs about which approach is best was a prominent discussion topic during the symposium, and remains a point for debate in order to inform integrated care policy and management in Ontario and elsewhere.

### Tension #3: Pay Inequities and Health Human Resources

One clear challenge that arose through symposium discussion related to the difficulty faced by home and community care organizations in maintaining a robust and highly trained cadre of health human resources. Although many outstanding health care providers choose to work in the home and community sector, the higher wages and more favorable working conditions in hospitals and primary care draw health care providers out of community settings. The challenge is exacerbated in home and community care by the difficulty in retaining personal support workers (paid carers who work at the homes of individuals needing help to remain independent). There is no simple solution to these challenges, particularly under resource constrained circumstances in which health systems find themselves around the world. However, identifying novel models of staffing and deployment, and opportunities to enhance the pay and improve the conditions of work for home and community care service providers, was deemed an important tension in the effort to implement the policy reforms of focus in this paper.

### Tension #4: Data Availability to Inform Improvement

The suggestion that data should be better used to understand practice patterns and outcomes according to the Triple Aim had clear consensus among symposium participants. However, participants also raised the issue of the wide variation in how organizations collect data for internal purposes, and the inconsistent collection of performance data by health system planners (i.e., the Local Health Integration Networks and the Ministry of Health and Long-Term Care) across sectors of the health system. The lack of capacity to systematically collect meaningful data, lack of infrastructure, and lack of new resources for such activity were identified as clear barriers to moving this agenda forward. Infrastructure for data collection and analysis is also something that requires new resources to produce, raising the issue of how resources might be allocated across a number of pressing health system needs that participants considered to be prerequisites to effective implementation of integrated care policy.

## Discussion

The recommendations and tensions identified from the symposium dialogue are highly relevant for the effort to promote collaborative governance in the context of an evolving policy environment. Each of the recommendations relates to particular governance strategies that are important for integrated care. However, the tensions raised illustrate that there are particular starting conditions that need to be acknowledged and a number of remaining conceptual issues to be addressed for a truly collaborative approach to governance of integrated care to be achieved in this policy setting. In this discussion section, we outline the ways in which the recommendations arising from the symposium could be strengthened by incorporating a more explicit approach to collaborative governance based upon the framework outlined by Ansell and Gash [3]. We then describe the unique challenges posed by an environment of policy change in the effort to achieve collaborative governance for integrated care, with reference to the situation in Ontario.

### Contributions to our Recommendations

In relation to the findings of our symposium on implementing integrated care in Ontario, Ansell and Gash [3] highlight several key points that could further advance our recommendations (see Table 1). First, by illustrating the importance of power or resource imbalances and the historical conditions in which these are situated, they emphasize the importance of acknowledging the possibility of institutionalized tension before the collaborative process begins. In relation to integrated care, this would suggest that discussing the challenges arising from potential imbalances between sectors of health and social care systems (e.g., funding imbalance between acute care and social care) could promote more positive dialogue and collaboration.

**Table 1:** Collaborative Governance: Enhancing the Symposium Recommendations.

Symposium Recommendations	Alterations from Collaboration Governance

**Recommendation #1: Enhance approaches to governance and accountability at the LHIN and sub-region levels to promote a more integrated patient experience of the health care system**	

1.1 Encourage shared accountability arrangements between health care delivery organizations wherever possible, in order to enable more integrated patient experiences of the health care system. 1.2 Establish clinician-level accountability mechanisms for more integrated care. 1.3 Develop incentives to build collaborative relationships with non-health system stakeholders, in order to connect patients with all the services they need.	**Starting Conditions:** Collaborative governance involves an explicit recognition of **power or resource imbalances** and **historical dimensions** that influence whether and how current collaboration unfolds. These dimensions are important considerations for any novel approaches to sharing accountability agreements and incentives acting on stakeholders. **Institutional Design:** Collaborative governance suggests that clear **ground rules** for engaging in collaborative work that do not unduly privilege any party drive the success of collaborative governance approaches. Such ground rules would be an important part of an approach to shared accountability.
**Recommendation #2: Establish metrics and measurement strategies that provide a clear picture of quality across the continuum of care, and reflect the perspective of patients, families and providers**	

2.1 Patients and caregivers should be systematically engaged to help co-design priority metrics that can be used to guide the implementation of Patients First. 2.2 Build health care providers perspectives and experiences into the evaluation of Patients First. 2.3 Enable provider and manager access to performance data relevant to their local level of care delivery.	**Intermediate Outcomes:** Collaborative governance suggests that “**small wins**” are an important component of the success of collaborative initiatives. Selecting metrics that can represent short-term successes would be an important addition to the development of metrics for integrated care in our recommendations.
**Recommendation #3: Leverage the sub-regions to enable health care providers to develop, scale and spread innovative strategies of care delivery**	

3.1 Identify and share best practices for engaging health care providers in the local development of innovative initiatives, including care coordinators. 3.2 Build clinical leadership at the sub-region level. 3.3 Streamline administrative functions to make innovation easier. 3.4 Build on innovative funding models that promote innovation, and particularly those in the areas of digital and mobile health. 3.5 Develop a provincial communications plan that emphasizes provider opportunities for innovation.	**The Collaborative Process:** Collaborative governance outlines the centrality of **trust building** to successful collaboration for complex problems, suggesting that face-to-face meetings are essential to building trust. In order to accomplish the goals identified in these recommendations, stakeholders must **meet face-to-face** and **commit to the process** outlined in order to successfully share best practices. Doing so is the primary mechanism by which **shared understanding** is achieved. **Facilitative Leadership:** Strong leadership that encourages a balance of perspectives and participation is seen to promote successful collaborative governance. Identifying individuals to explicitly **lead the collaborative process** will support successful collaboration.
**Recommendation #4: Continue to engage patients and caregivers as central partners in health system planning**	

4.1 Continue to enable patient and caregiver engagement at the level of the LHINs and sub-regions. 4.2 Support training and capacity development of patients and caregivers. 4.3 Develop a communications strategy specifically directed to patients and the public that tells the story of how the health care system is changing, why, and what will be different for them as users of the system.	**Institutional Design:** To the extent that patients’ views should be incorporated in the collaborative governance process, the involvement of patients must be a **systematic element of the institutions** in which collaborative governance takes place.

Second, Ansell and Gash [3] describe the importance of clear ground rules governing the actual processes by which people from different organizations or sectors interact in the collaborative process. By ruling particular topics or issues in or out of discussion, clear ground rules can further specify the targets of conversation and enhance goal-oriented collaboration across stakeholder groups for more integrated care.

Third, the value of intermediate outcomes as a motivating influence for challenging collaborations is explicitly acknowledged in the collaborative governance model. By selecting metrics that allow for “small wins” early in the collaborative process, leaders can better promote a positive orientation toward the collaboration among all stakeholders involved.

Fourth, Ansell and Gash (2008) clarify the importance of face-to-face meetings in order to build trust. This point was overlooked in the symposium recommendations, and we acknowledge this is central to achieving successful collaborations for integrated care. Face-to-face meeting demonstrates commitment to the collaborative process and represents an important strategy to generate shared understanding among stakeholders.

Fifth, a specific leader is identified in the model of collaborative governance to spearhead and coordinate the collaborative initiative. Although individual leaders tend to emerge throughout the course of implementing integrated care and quality transformation [19], explicitly identifying this person (or small group of people) a priori can help to further clarify the collaborative process. Where challenges arise during the collaboration, the leader is then able to facilitate solutions and prioritize group cohesion.

Sixth, whereas our recommendations have recognized the importance of systematically incorporating patient experiences and views, the collaborative governance model recasts this issue as one of potential institutionalization. What processes and values have been institutionalized, through particular policies and rules that might interfere with authentic patient engagement [20]? By highlighting that patient engagement must be thoroughly institutionalized by being built into the policies and procedures for integrated care initiatives, the collaborative governance model points toward new avenues for patient engagement beyond the ad hoc inclusion of patients and caregivers in sporadic program decision-making.

In summary, the model of collaborative governance outlined by Ansell and Gash (2008) encourages researchers and practitioners to view the effort to achieve integrated care through a broader framework that acknowledges the many influences on collaborative behaviour involving governing bodies in the public sector. These enhancements to our recommendations should help to inform the effort to engage managers and clinician leaders in the generation and implementation of integrated care initiatives, highlighting the important roles they can play in promoting stronger collaboration at the governance level across stakeholder groups. Though the recommendations and tensions discussed in this paper are for managerial and clinician audiences, policymakers play a central role in mitigating barriers to their implementation by promoting policies which enable collaboration. This requires policymakers to review and amend current policies to support these initiatives and to be mindful of these issues while drafting future policies.

### Collaborative Governance and Policy Change

In this paper we have presented the key features of an evolving policy environment, and presented a set of recommendations for ways in which health care leaders can promote the implementation of more integrated care using collaborative governance strategies in a changing context. Health system stakeholders in Ontario are currently determining how to proceed with their ongoing efforts at implementing more integrated care as the current government puts into place its new policy objectives. Drawing on the collaborative governance framework, we identify two challenges to the effort to produce collaborative governance posed by the policy changes in Ontario, and suggest that these challenges apply more broadly to the effort to implement integrated care in any changing policy environment.

The first challenge posed by the changing policy environment in Ontario is the lack of any consistent institutional design in which collaborative governance could take place. The institutions governing collaboration stand to change quite drastically as the details of the new policy become clearer, and teams have been forced to question existing collaborative arrangements as a result. As the new policy institutes a new funding mechanism, teams will need to re-visit the work they have already accomplished regarding aligning resources to deliver more coordinated care. As the existing institutional framework for supporting integrated care changes, so too will organizations’ strategies for collaborating with other organizations. The changes to the institutional environment present yet another contingency to be considered by health care leaders as they work to advance a more integrated agenda.

The second challenge arising from the environment of policy change relates to the availability of stable facilitative leadership. The collaborative governance model specifies that public actors are the group who initiate collaborations that benefit from collaborative governance arrangements and often take on the facilitator role. With the dissolution of the Local Health Integration Networks that is mandated by the new policy in Ontario, the public actors who had been providing that facilitative leadership will no longer be available to support collaborative efforts. Furthermore, it is unclear whether those supporting collaboration through facilitative leadership are being eliminated as a resource-saving measure, or whether they will reappear in a new form when the dust settles. These observations raise the important question: Where will facilitative leadership come from to support collaborative governance in ongoing collaborative arrangements?

## Conclusion

In this paper we have presented a set of recommendations and tensions arising from a policy stakeholder dialogue on the implementation challenges associated with integrated care policy in Ontario. We examined the recommendations from the perspective of collaborative governance, identifying a number of actionable strategies to enhance the recommendations inspired by a collaborative governance approach to integrated care. The two challenges we identify here pertain especially to the realities of implementing integrated care when political parties change in a continuous cycle in many governments around the world. Political parties determine policy, and policy stipulates the funding and health system structures in which health care leaders and providers must deliver health services.

These observations lead to a concrete recommendation for policymakers as they move to enact health policy changes that could be highly disruptive for the ongoing work of making integrated care a reality: Consider the ways in which any new policy change might disrupt elements of collaborative governance that are truly essential for integrated care, such as institutional arrangements that encourage collaboration and the availability of leadership to facilitate change. Although these insights have been addressed before in literature on the governance of integrated care [21,22], this is the first we have seen them highlighted specifically in the context of a broader understanding of the collaborative governance arrangements that might better support integrated care. Where policymakers are able to better anticipate the impacts of policy change on these important features of collaborative governance, stakeholders can be better prepared to ensure the necessary conditions are in place to maintain successes already achieved in building partnerships for new models of integrated care. We believe this is feasible and would enhance the ways in which integrated care policy can be understood and adopted by health care stakeholders.
